# Determinants of the utilisation of sexual and reproductive healthcare services by male adolescents in the Tshwane Metropolitan Municipality in South Africa

**DOI:** 10.4102/hsag.v24i0.1203

**Published:** 2019-11-11

**Authors:** Omari Shabani, Takalani G. Tshitangano

**Affiliations:** 1 Department of Public Health, School of Health Sciences, University of Venda, Thohoyandou, South Africa

**Keywords:** determinants, healthcare services, male adolescent, sexual and reproductive health, utilisation of sexual and reproductive healthcare services

## Abstract

**Background:**

Male adolescent sexual and reproductive health is one of the essential healthcare programmes in the world. However, male adolescents still face numerous challenges in this area. Determinants of the utilisation of these services need to be known to develop strategies to improve utilisation of the available services by male adolescents.

**Aim:**

The aim of this study was to identify the determinants of the utilisation of sexual and reproductive healthcare services by male adolescents in the Tshwane Metropolitan Municipality in South Africa.

**Setting:**

The study was conducted in the Tshwane Metropolitan Municipality in South Africa.

**Methods:**

An explorative, descriptive and qualitative approach was employed. The study was contextual in nature, and purposive sampling was used. The population of the study consisted of male adolescents (aged 18–24 years) living in the Tshwane metropolitan Municipality. Twenty male adolescents participated in the study. Data collected using semistructured individual interviews were analysed using Tesch’s method of data analysis, and measures to ensure trustworthiness and ethical consideration pertaining to the study were established.

**Results:**

The perception of existing services was found to be a significant individual factor influencing them negatively in utilising the services. This was linked to the violation of their rights as human beings by healthcare providers, their unmet expressed needs and the ineffectiveness as well as inefficiency of the support structures.

**Conclusion:**

These results suggest that utilisation of these services by male adolescents can be improved by changing their perception of the existing services through support from different structures of the society.

## Background information

Sexual and reproductive health (SRH) is a state of complete physical, mental and social well-being in all matters relating to the reproductive system (Pan American Health Organization [PAHO] 2010). In order to reach this state of completeness, people should be able to have a satisfying and safe sex life, the capability to reproduce and the freedom to decide if, when and how often to do so. To maintain one’s sexual and reproductive health, one needs access to accurate information on safe, effective, affordable and acceptable contraception methods of their choice. People must be informed and empowered to protect themselves from sexually transmitted infections. All these can be achieved through the effective and efficient utilisation of sexual and reproductive health services (SRHCS) available or at their disposal.

Sexual and reproductive health is also about human rights for both men and women. With regard to these rights, there is much literature calling for the need for programmes for males to be put in place in South Africa as there is little (or nothing) in the way of policies or programmes that have specific guidelines for male SRHCS (Ramkissoon et al. 2010). According to Collumbien and Hawkes (2010), researchers have observed the failure of public sector programmes to take cognisance of male sexual health problems. This fact is probably one of the factors that may lead them to continue seeking care for all their sexual health problems in the unregulated and possibly ineffective private sector. If programmes addressing the sexual health needs of males are to be effective, they will need to be comprehensive in their scope and coverage, just as they are now aiming to be for women (Collumbien & Hawkes 2010).

It has been documented that sexual and reproductive healthcare services are usually poorly utilised by adolescents (Binu et al. 2018). Some of the factors identified to be responsible for this are: lack of privacy, inconvenient clinic times, religious reasons, cultural factors, parental monitoring and communication, number of sexual partners, duration of romantic relationships and access to information from other sources like peers and school teachers (Binu et al. 2018; Felekke et al. 2013).

### Definition of terms

The concepts that are central to this study are defined as follows:

*Adolescence* is the transitional phase between childhood and adulthood (PAHO 2010). In this study, adolescent refers to males who are aged between 18 and 24 years.

*SRHCS*: Aim at the achievement and promotion of both sexual and reproductive health by public healthcare providers to the community to reach a state of physical, mental and emotional well-being in all aspects related to sexuality and reproduction.

*Utilisation of SRHCS*: Refers to the ability to access and make use of, in an appropriate and convenient manner, the SRHCS that are available in a particular area. It is the use of healthcare services by people (Awoyemi, Obayelu & Opaluwa 2011).

### Problem statement

According to WHO (2014, 2015) and UNAIDS (2016), adolescents and youth have the highest incidence of HIV and AIDS-related conditions today. Despite the availability of sexual and reproductive healthcare services in South Africa, adolescents in general, and male adolescents in particular, are still experiencing many challenges in this area. Some of the challenges include teen pregnancy and the prevalence of HIV and AIDS (5.5%) that affect both male and female adolescents (Statistics South Africa 2018). However, most intervention programmes are focussed more on female than male adolescents in their planning and execution, thereby rendering the males passive recipients of such services. This is supported by the findings of Ramkissoon et al. (2010) who state that the sexual and reproductive health needs and the rights of men are generally not addressed in the public sector health services where male reproductive health services are largely absent.

The Tshwane Metropolitan Municipality has a high prevalence of HIV (11.7% in 2012) and the researcher has observed a lack of sexual and reproductive healthcare services designed specifically for males as well as male adolescents’ under-utilisation of available sexual and reproductive healthcare services. Male adolescents are not often mentioned as an ‘at-risk group’, and there is a scarcity of studies on male adolescents and their utilisation of the available SRHCS. The researcher holds a view that focus should be on male adolescents, educating them appropriately and ensuring they use available facilities so that they are empowered to become responsible adult males. This is the motivation behind the study.

### Significance of the study

This study may assist the policy-makers to identify and understand the factors that influence male adolescents’ utilisation of sexual and reproductive healthcare services and thus help them in the development of policies that encourage and support male adolescents in the uptake of the services. It may also be of great benefit to health personnel to discover their indispensable roles in the welfare of these adolescents and execute their duties in a more youth-friendly manner. If these policies are enforced, male adolescents can be expected to have a better access to healthcare services that address their sexual and reproductive needs, which would help them to subsequently lead healthier lives that will eventually impact positively on their lives.

## Methods and materials

### Research design

An exploratory, descriptive and qualitative design was used to conduct the study (Creswell 2013), as the researcher attempted to gain more facts and a broad understanding of the factors influencing the participants’ utilisation of the services. The study was contextual in nature. Qualitative approaches allowed the researchers to interact with participants in order to gain a deeper understanding of the phenomenon being studied and to view the findings in the context of the respondents’ worldview (Polit & Beck 2012).

### Setting

This study was conducted in the Tshwane Metropolitan Municipality. This was chosen because of the following reasons: HIV prevalence rate (11.7% in 2012) in the Metropolis, lack or scarcity of statistics related to male adolescents’ utilisation of these services, lack of services dedicated to males, lack or scarcity of evidence-based studies on male adolescents and their utilisation of these services and in most cases, male adolescents are ignored in sexual and reproductive-health-related matters and discussions. The assumption is that male adolescents are an at-risk group.

### Population and sampling

The study population consisted of male adolescents (aged 18–24 years) living in the Tshwane Metropolitan Municipality. Purposive sampling technique was used (Polit & Beck 2012) to recruit participants who met the inclusion criteria (being a male adolescent living in the designated area, being 18–24 years old and willing to participate in the study). The researcher involved colleagues, friends and supervisors to identify and/or refer potential participants. The recruitment was done by means of telephone calls to set up appointments for briefings as well as the interviews. Because of the difficulty of getting the research participants, the briefing sessions were not done in a group but with each and every participant separately. During the sessions, the participants were informed of the aim, objectives of the study, their legal rights and what would happen to the data (information) they would provide; they were also requested to sign the informed consent form, indicating their willingness and acceptance to participate in the study. The total number of participants who took part in the study was 20. This number was informed by data saturation (Data saturation was reached after the 15th respondent could not provide any new information relevant to the aim and objectives of the study. However, the interviews continued until the 20th respondent).

### Data collection

Data were collected by means of semi-structured individual interviews between January and August 2016. The interviews were conducted in English to avoid interpretation- and translation-related challenges. Data were collected in a assigned room in the clinic, which was normally used for counselling clients. The room was private, and it did not raise any suspicions, as it was a normal clinic room. Semi-structured individual interviews that lasted between 30 and 45 minutes were conducted. Interviews were audiotaped with the participants’ permission. A common question asked was: ‘Tell me what influences utilisation of SRHCS by male adolescents?’ Many probes followed this question depending on the responses. This was in accordance with the guidelines proposed by Hennink et al. (2011). Examples of probing questions included the following: ‘Can you tell me more? What do you mean? What pushes you to utilise these services? What stops you from utilising these services?’

### Data analysis

Data collected in the field in the form of voice recordings were transcribed verbatim and later analysed using Tesch’s method of data analysis, which consists of eight steps, involving separating, examining, comparing and categorising raw data with the purpose of amalgamating it in a new way (Creswell 2013). Two researchers, independent of one another, completed the process of data analysis utilising the Tesch’s method of data analysis. The third researcher checked a sample of four transcripts to verify their correctness. Data were listed and grouped into preliminary groupings of descriptive themes agreed upon by the three researchers during a consensus discussion meeting.

### Trustworthiness

Trustworthiness of the study was established by ensuring that credibility, transferability, dependability and confirmability were explored. These were ensured through the adoption of well-established research methods, the provision of detailed description of the research process, the use of purposive sampling technique, the documentation of real-life experiences and real personal stories of participants, and the substantiation of the report of the interviews by reviewing similar studies previously conducted. Reliability of the data was ensured by involving all authors in the transcription and cross validation of the data. The researcher reflected on the impact of his role, personal background and culture on the study before, during and after the study, and has prevented and avoided the occurrence of any possible biases.

### Ethical considerations

Ethical Clearance was received on 20 November 2013. Ethical clearance (HSHDC/245/2013) was sought from and granted by the ethics committee of the Department of Health Studies of the University of South Africa. Permission to conduct the study in the Tshwane Metropolitan Municipality was also sought from and granted by the Tshwane Metropolitan Municipality (Health and Social Development Department Multisectoral AIDS Management Unit). All the participants signed the consent form before data collection, indicating their willingness and acceptance to take part in the study. The following ethical considerations pertaining to the study where adhered to: informed consent; voluntary participation; confidentiality and participants’ rights to autonomy, self-determination, privacy, fair treatment and avoidance of harm; As well as the researcher’s responsibility to seek advices.

## Findings

### Socio-demographic characteristics

Out of the 20 purposively selected participants, five were 19 years old, six were 20 years old, two were 21 years old, three were 22 years old and four were 23 years old. Amongst them, 18 participants were students while two were not. All participants were fluent in English.

### Emerged themes

Four major themes emerged from the analysis of the collected data, namely, male adolescents’ perception of existing SRHCS, violation of their rights as human beings, male adolescents’ expressed needs and support structures.

### Male adolescents’ perception of sexual and reproductive health services

Participants indicated that the lack of information about the services, nurses/healthcare providers’ attitudes, staffing in services and the quality of the services negatively influenced their perception of the services. This is what they said:

‘I think it is poor because of we as youth are scared to go to clinics and government hospitals for information because they always judge you. It is not easy to communicate with them and ask for information … (paused) yes … (paused) basically what I am saying you can’t go and communicate with them because they always judge and you get the information that you want …’ (P1, 22 years, male, student)

### Violation of their rights as human beings

Participants indicated that because of the nurses’ attitudes, lack of respect towards them, unprofessionalism and being denied easy access to the services, their rights as human beings were violated. They also felt stigmatised and discriminated against at these services. Stigmatisation, as used in this study, means the display of nurses and/or healthcare providers’ negative and judgmental attitudes towards them as well as refusal to give them proper attention, care and treatment. This is what they said:

‘I really do not know how to say it but my observation is that there are better services in private clinics than in public clinics and hospitals because of the way the staff treat you. In private clinic, they treat you with respect, dignity and care, in fact like a king. But in public clinics, they see you like a piece of some dirty thing …’ (P11, 19 years, male, student)

Participants considered the lack of services designed specifically for them, the availability of services only designed with females in mind and the lack of/or no consultation by stakeholders in matters regarding their SHR issues as gender discrimination and marginalisation. This made them feel frustrated, discriminated against, marginalised, neglected, with no consideration whatsoever as human beings with needs and desires. This is what they said:

‘I think we men are not really considered as human beings. You know even when people talk about social grants; do you know it is not given to men, only to women? Is this not another form of discrimination or marginalization? So men, don’t have the rights to treatment and other social benefits.’ (P15, 21 years, male, student)

### Male adolescents’ expressed needs

Four sub-themes emerged from this theme, namely, therapeutic relationships between nurses and the patients; government intervention in service delivery; inclusive programmes and advocacy for integrated school services; and information needed about the services.

#### The therapeutic relationships between nurses and male patients

This has been impaired by the nurses’ attitudes. This then evoked negative feelings and emotions in the adolescents, some of whom said:

‘… The healthcare professionals, especially nurses are not professional at all. They do not respect you, they judge you, and they do not care at all.’ (P11, 19 years, male, student)

#### Government intervention in service delivery

Participants expressed their desire for the government to be fully involved in the service delivery through: employment of adequate staff to cater to their needs; making sure that the services are accessible, reliable, effective and sustainable; as well as development of strategies that enable, motivate and encourage them to utilise the services without any fear or reservation of any kind. But, more importantly, to involve them and their parents in decision-making processes concerning their SRH challenges. This is what they said:

‘There should be a section to deal with only males and not being mixed with girls, the government needs to train and employ male nurses to treat us not female ones because these are the people who stigmatize and discriminate against and us.’ (P11, 19 years, male, student)

#### Inclusive programmes and advocacy for integrated services

They also expressed their desire to have these services integrated into the school curriculum and the establishment of SRHCS in their schools. This is what they said:

‘I would suggest that these services be offered at school because that’s where we spend most of time and the school or the university must make sure that all the students, especially males know about these services and what they offer and encourage the students to use them on a regular basis … if the services can be fully integrated in some subjects like Life Orientation so that we can start learning these issues on a regular basis and even write tests and exam … it will be a good think.’ (P19, 20 years, male, student)

#### Information needed about the services

Participants also expressed their desire to have access to information on SRHCS. This is what they said:

‘For example, creating a platform, even a call center where parents can share their experiences on how to provide sexual and reproductive health information to their children. I am saying because many parents are still reluctant may be because of their cultural and religious beliefs, to talk to their children about sex because in their culture it is a taboo … or because they said this and that … I think that platform will give the parents the opportunity to engage with their peers about cultural and religious constraints and be able to open to their children.’ (P17, 23 years, male, student)

In addition, participants indicated their willingness to be fully involved in and take ownership of any activity aimed at the prevention, reduction and treatment of sexual and reproductive health issues including HIV and AIDS within the school and the community. Sexual and reproductive health services through the government’s channels need to ensure that the youth in general and male adolescents, in particular, are aware of these services. This is what they said:

‘I think if you want to promote the utilization of these services among us male adolescents, my idea would be to get the male adolescents involved in the campaign programmes. It is also important to go to schools and university because some of us are already at university while some of our age mates are still at schools to encourage us to take part in the programme.’ (P18, 20 years, male, student)

### Support structures

Support structures include the government and all stakeholders involved in the sexual and reproductive healthcare services which can influence male adolescents to utilise the services. Two sub-themes emerged from this theme, namely, the roles of other stakeholders and government interventions. The following groups were identified as stakeholders: parents; schools; churches; mass media; and social media. Participants seek the support of the above-mentioned groups regarding their utilisation of SRHCS. This is what they said regarding the role of government:

‘The government needs to come up with a strategy on how to encourage parents like my father who is so tied to their culture to soften and become flexible and start talking about sex with their children because even though and because of my age, I am still not yet involved in sex (P18, 20 years, male, student) … ‘May be the government needs to tell our parents and the school when to start teaching us about sex, how to prevent diseases and how to act responsibly in life (P18, 20 years, male, student)’. Let the government use all the means and ways possible to make sure we have the necessary information regarding these issues. Let the government involve us in whatever it is doing to enhance our active participation. Let the government bring the services to us and to use walking distances looking for services.’ (P17, 23 years, male, student)

#### The roles of stakeholders

Participants indicated that, given their roles in the society, parents, churches and schools should be fully involved in SRHCS, and these services should be integrated into the school curriculum to enhance its utilisation for the betterment of their lives. This is what they said:

‘Like at a church whereby we talk about the use of condoms if the priest say we should not use or that, what about the medical side? Condoms or distribution and young people having abortion as in primary care actually, we don’t have an expert, that might seem as if promoting sexuality but then what about the medical side of the story? That might be seen as promoting sexual activities, we don’t have anyone who knows anything about the topic we don’t have an expert to talk about these issues but they become more involved in these kind of programmes … just like a normal meeting like what we have in church, get a health expert to get the youth event and address these issues just like in normal meeting to the church, … they have them in this major event once a year … then they can organize for the youth to come and talk to them.’ (P2, 20 years, male, student)

Participants indicated that mass media, such as TV, radio, newspapers and others, can be used in the prevention of SRH challenges, including HIV and AIDS. The use of the mass media has been reported to be an effective and efficient tool in informing and educating people regarding so many social phenomena, and HIV and AIDS is no exception. This is what they said:

‘Yes, they can use the media especially … especially social media and the information should target more males than females because currently the information is more targeted to females … no, you bring the services to them, they don’t go to them, the services come to them and not wait for them to come to the services when there are events such social events, make sure that those people are there.’ (P2, 20 years, male, student)

## Discussion

The participant’s perception of the existing services was found to be a significant individual factor influencing them negatively in utilising the services. This was linked to violation of their rights by healthcare providers, their expressed needs and the ineffectiveness as well as inefficiency of the support structures. These findings confirm the findings of Ayehu, Kassaw and Hailu (2014); Kaufman et al. (2016) who reported that, adolescents’ utilisation of sexual and reproductive services is limited owing to judgmental attitudes of service providers and structural factors. It was found that, in the absence of effective education, consultations with peers on the issues increased responsible sexual health decision-making, but resulted in transfer of erroneous information and reinforcement of risky sexual practices by males (Kaufman et al. 2016). It was also revealed that Ghanaian adolescents still avoid SRH services, owing to the negative of the health providers (Aninanya et al. 2015). Access to services, gender requirements for treatment within the facility, fear in anticipation of the services provided and having concerns about confidentiality were barriers to SRHCS (Bender & Fullbright 2013). The health providers’ negative attitudes towards adolescents were evident in a number of studies as a hindrance to accessing SRHCS (Chilinda et al. 2014; Kaufman et al. 2016). Thus, poor attitude was reflected by the judgmental approach of the health providers. Adolescents reported that service providers in normal clinics treat them rudely or deny them services (Chilinda et al. 2014; Kaufman et al. 2016). As a result, young people feel neither well-received nor comfortable in mainstream family planning clinics. In a South African study conducted among nurses, it was reported that the nurses generally stigmatised adolescent sex and felt very uncomfortable giving contraception to adolescent girls; they often tried to influence the adolescents who came for contraception not to have sex (Chilinda et al. 2014; Kaufman et al. 2016). Parental permission was also sought from adolescents before contraceptive services were provided even though legally, parental permission is not needed for minors to be given contraception in South Africa (Chilinda et al. 2014; Kaufman et al. 2016).

Participants indicated being stigmatised and discriminated against by service providers in general, and family planning and HIV clinics. This finding is in line with the findings of Coleman et al. (2016); Harper, Lemos and Hosek (2014); Kerr et al. (2015) who revealed that HIV-related stigma, which is a significant impediment to combating the HIV and AIDS epidemic, constitutes a key factor impeding HIV identification, prevention and treatment. Practice as a healthcare professional is based upon a relationship of mutual trust between patients and healthcare practitioners. To be a good healthcare practitioner requires a life-long commitment to sound professional and ethical practices and an overriding dedication to the interests of one’s fellow human beings and society. In essence, the practice of healthcare professions is a moral enterprise (Health Professional Council of South Africa 2008).

Participants also expressed the desire to see the government employing adequate staff to cater to their needs, ensuring the reliability, effectiveness and sustainability of the services. But more importantly, they desired the involvement of male adolescents in decision-making processes concerning their SRH challenges. This being the case, they would really like to take ownership and lead the processes. This finding is supported by the findings of Villa-Torres and Svanemyr (2015) who argued that young people have a fundamental human right to participate in matters that affect their lives. Giving decision-making power to young people and integrating them into all aspects of programme development are vital components of ensuring meaningful participation (Villa-Torres & Svanemyr 2015). Simply having youth programmes within an organisation does not necessarily guarantee meaningful youth participation. Several conceptual frameworks have been developed to measure youth participation, but more innovative approaches to effectively engage young people in SRHR programmes are needed (Villa-Torres & Svanemyr 2015). In addition, it is argued that school-based health clinics, or school-based health centres (SBHCs), are considered to be one of the most effective strategies for delivering comprehensive primary and preventative health services to young people, especially those that are normally underserved by health services (Manson-Jones et al. 2012).

Access to services and accurate information about prevention against HIV and AIDS and reproductive health for young people is problematic. In South Africa, a number of reports have documented the poor quality of care that young people receive in the public health services. Throughout southern Africa, this poor quality of care for young people encompasses limited access to services, poor reception and treatment from service providers and non-availability of preventive methods that young people need, most notably condoms (Karim & Karim 2010). Furthermore, the provision of accurate and specific information is an important primary goal for intervention programmes (Karim & Karim 2010). Providing young people with SRH information and services through the existing healthcare system presents an opportunity that should be further optimised (Godia et al. 2014).

Adolescent–parent SRH communication was largely limited among adolescents who had poor behaviour, beliefs subjective norms to communicate on sexual issues with the parent, and perceived the parents had poor SRH knowledge. Nonetheless, high adolescent–parent general communication quality, television coviewing and discussion, and adolescent self-disclosure substantially improved the communication. Therefore, engaging parents in sexual education of the adolescents, improving underlying beliefs and norms, and improving the adolescent–parent communication, self-disclosure, television coviewing and discussions are essential (Dessie, Berhane & Worku 2015).

The church has a role to play in shaping the sexual and reproductive life of male adolescents. In South Africa, Christian faith communities exert a powerful influence on attitudes and life styles, and have credibility in the society, and perhaps that is one of their major assets. Furthermore, local churches are present in both urban and rural areas, and their extensive networks can be valuable in delivering health services such as HIV prevention to young people. By providing individuals with education, rules, rituals and social networks among peers as well as across generations, the local faith communities create a structured social environment where young people can be socialised. The churches may serve as socialising entities for those youth who attend religious services and can provide them with a sense of belonging, which is important during adolescence. In general, religion is described as a protective factor for young people with regard to sexual behaviour and can be associated with behaviours such as delayed sexual debut, lower likelihood of voluntary sexual activity and fewer sexual partners outside romantic relationships (Eriksson et al. 2014).

The judicious use of media is a promising approach to addressing sexual and reproductive health issues, including HIV and AIDS, to demystify HIV disease. Innovative, multifaceted media approaches have increased the visibility of HIV as a health concern and successfully promoted better preventive behaviours (Kerr et al. 2015). Project iMPPACS, a culturally tailored, multifaceted (radio, television), mass media campaign, demonstrated effectiveness in reducing HIV risks among African American adolescents in multiple cities (Karim & Karim 2010). In China, mass media sources, such as TV programmes, newspapers, and magazines, were among the most frequently identified channels for HIV information. People who were exposed to mass media were more knowledgeable about HIV and stigmatised less those persons living with HIV and AIDS (Jesmin, Chaudhuri & Abdhullah 2013). Mass media interventions have the potential to reach large audiences, providing them with information and raising awareness. They may use broadcast media, such as television, radio or film; print media, such as posters and newspapers; outdoor media, such as billboards; or digital media, such as the internet (French et al. 2014). Since the beginning of the HIV epidemic, mass media programmes have proved to be important in increasing knowledge and promoting preventive practices (Do, Kincaid & Figueroa 2014). Sommer and Mmari (2015) argue that the media are a powerful force on Adolescent Sexual and Reproductive Health (ASRH). According to a recent literature review on the social and emotional factors that influence condom use among adolescents in Lower Middle Income Countries (LMICs), when adolescents were exposed to mass media (radio, television, the internet), they were much more likely to use condoms (Sommer & Mmari 2015).

Participants indicated that social media, such as internet, face-book, twitter and others, can be used to convey the information and messages to the youth in general and to male adolescents, in particular, regarding sexual and reproductive health issues including HIV and AIDS and its impact on them (Jaganath et al. 2012). Social media platforms, including mobile technologies and social networking sites, are being used increasingly as part of HIV prevention and treatment efforts. Importantly, social media provides users with the opportunity to generate, share and receive information through bi- and multidirectional exchanges, which may transcend geographic borders and provide an opportunity for anonymity (Taggart et al. 2015). Also, the internet can facilitate HIV prevention among a subset of men who have sex with men by enhancing awareness, service uptake, retention in care and adherence to treatment (Cheng et al. 2016).

## Limitations of the study

The small size of the sample limits generalisation and external validity of the findings as well as the scope of the research. The transferability of the findings is limited owing to the data being gathered in only one area.

## Recommendations

The study recommends the development of strategies to improve utilisation of sexual and reproductive healthcare services by male adolescents. Such strategies include training of youth-friendly service providers specifically on the sexual and reproductive health needs of male adolescents, employment of these trained individuals in health facilities, regular health education for male adolescents, and counselling and provision of contraceptives. Community support can be mustered for male adolescents through organising community sensitisation campaigns and empowerment of male adolescents to actively participate in addressing their own sexual and reproductive health needs. These measures could help in preventing, reducing and eradicating SRH challenges experienced by male adolescents on a daily basis.

## Conclusion

Utilisation of SRHCS by male adolescents can be improved by changing their perception of the existing services through support from the society, meeting their needs and respecting their rights. This implies the development of strategies to improve utilisation of SRHCS for prevention, reduction and eradication of their SRH challenges.
